# Negative Effects of Paternal Age on Children's Neurocognitive Outcomes Can Be Explained by Maternal Education and Number of Siblings

**DOI:** 10.1371/journal.pone.0012157

**Published:** 2010-09-14

**Authors:** Ryan D. Edwards, Jennifer Roff

**Affiliations:** 1 Department of Economics, Queens College and the Graduate Center, City University of New York, Flushing, New York, United States of America; 2 National Bureau of Economic Research, Cambridge, Massachusetts, United States of America; University of Cambridge, United Kingdom

## Abstract

**Background:**

Recent findings suggest advanced paternal age may be associated with impaired child outcomes, in particular, neurocognitive skills. Such patterns are worrisome given relatively universal trends in advanced countries toward delayed nuptiality and fertility. But nature and nurture are both important for child outcomes, and it is important to control for both when drawing inferences about either pathway.

**Methods and Findings:**

We examined cross-sectional patterns in six developmental outcome measures among children in the U.S. Collaborative Perinatal Project (n = 31,346). Many of these outcomes at 8 mo, 4 y, and 7 y of age (Bayley scales, Stanford Binet Intelligence Scale, Graham-Ernhart Block Sort Test, Wechsler Intelligence Scale for Children, Wide Range Achievement Test) are negatively correlated with paternal age when important family characteristics such as maternal education and number of siblings are not included as covariates. But controlling for family characteristics in general and mother's education in particular renders the effect of paternal age statistically insignificant for most developmental measures.

**Conclusions:**

Assortative mating produces interesting relationships between maternal and paternal characteristics that can inject spurious correlation into observational studies via omitted variable bias. Controlling for both nature and nurture reveals little residual evidence of a link between child neurocognitive outcomes and paternal age in these data. Results suggest that benefits associated with the upward trend in maternal education may offset any negative effects of advancing paternal age.

## Introduction

The demographic transition has brought declining fertility rates and population aging across the industrialized world [1]. Simultaneously, increases in female education, labor force participation, and earnings have coincided with reduced family sizes and delayed marriage and childbearing [2]. The average maternal age at first birth in the US is now 25, up from 21.4 in 1970, although it is still the lowest among 14 other industrialized countries [3]. Relative to the annual gains in period life expectancy at birth, which have averaged 0.21 year in industrialized countries [4], an annual increase in age at maternity of 0.10 may seem relatively modest. But studies of trends in the age at menopause have revealed mixed results [5], and advanced maternal age may be associated with some adverse health consequences [6].

Assortative mating typically produces fairly tight correlations between maternal and paternal ages [7], and as a result, there is interest in whether advancing paternal age is important for child outcomes. An array of studies have shown advanced paternal age to be associated with neurological disorders, especially schizophrenia [8], [9], and a recent study [10] reveals a negative association between paternal age and children's neurocognitive outcomes in US data from the 1960s and 1970s. The study finds that advancing maternal age is relatively benign, while paternal age is associated with deleterious child outcomes, which the authors suggest may be related to the heightened mutation rate in sperm. A companion piece [11] discusses the implications and the robustness of the findings, focusing in particular on how the negative effect of father's age becomes somewhat attenuated once family socioeconomic status is controlled. This result is somewhat puzzling given that one would expect paternal age to be positively associated with family income and wealth, and for the latter to be positively associated with child outcomes. A negative impact of father's age on child development is also at odds with a recent paper highlighting the role of long male reproductive lives in the evolution of human senescence past the age of female menopause [12].

In this paper, we aim to inject a more rigorous discussion of family economics into the current inquiry of male biology and children's outcomes. Assortative mating suggests that delayed childbearing typically also coincides with higher paternal age, but we know that mates sort on education and other characteristics [7], and these patterns are not necessarily stable over time [13]. Given that childbearing is restricted to premenopausal years for females while it is unrestricted for males, any subsample of new children with older fathers is also likely to have mothers who are much younger than their mates. In US data, we find that mother's education can be negatively correlated with father's age. Education measures the quality of the mother's human capital [7], and studies have repeatedly shown that it improves child outcomes, presumably by raising the value of maternal inputs [14]–[17]. The omission of maternal education in an observational study is likely to produce biased results because it is correlated with factors like paternal age.

Research suggests that siblings compete for parental or household resources, and that parents face a fundamental tradeoff between the quantity of children they have and the quality of each child they can produce through investments of time and other resources [7]. Because of the inverse connection between quantity and quality, the number of siblings, like mother's education, is an important variable in understanding child outcomes, and it is also correlated with paternal age. When it is an omitted variable, number of siblings is likely to bias the relationship between father's age and child outcomes in a negative direction.

We intend our goal in this paper to be constructive in nature, highlighting the contributions of both nature and nurture, via the economics of the family, to child outcomes. We view previous contributions to this literature [10] as useful and thought-provoking. Our aim is to clarify the functional relationships between child outcomes and biological, socioeconomic, and behavioral factors by drawing on accumulated interdisciplinary knowledge.

## Methods

### Theory

Our perspective on early child development is that outcomes derive from natural and nurturing inputs alike. The former primarily comprise genetic endowments passed from parents to children. As previous research has suggested [8]–[11], parental age may be a good proxy for the quality of genetic material. Birth weight measures a child's initial health endowment and reflects both heredity and also prenatal nurturing.

Postnatal nurturing inputs include the quality and quantity of time spent by parents on the children, as well as food, clothing, and other elements provided by the family [7]. Other things equal, increased family size reduces time and money available for inputs on a per-child basis [7]. A large body of research [14]–[17] has examined child outcomes relative to parental inputs using household time diaries and other data. This literature shows that the quantity and quality of time spent with children increases with parental education, especially maternal, and child outcomes improve accordingly. When data on actual time inputs are not available, parental education is likely to be a good proxy of total nurturing inputs, and family size determines the amount of inputs available per child.

It is of course possible that maternal education is correlated with unobservable characteristics of the mother, such as scholastic ability, that affect child cognitive outcomes as well. In this paper, we do not attempt to draw causal inferences regarding the effect of maternal education on child outcomes. Rather, we examine the complex interrelationship between paternal age, maternal education, which may proxy for other characteristics, and child outcomes.

With measures of natural or hereditary factors 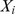 and nurturing inputs 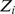 in hand, we seek to investigate the determinants of child development by modeling child 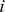
's outcome 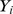 according to a standard reduced form:
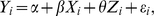
(1)where 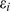 is a white-noise disturbance. In observational data, least-squares estimates of 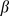 and 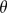 are at best only suggestive of causal relationships, and estimates may also suffer from bias. In particular, omitted variable bias will arise if 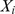 and 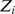 are correlated with 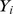 and with each other, and if either is omitted from the model.

### Sample

We examine the same data on child outcomes that were previously analyzed [10], which consist of developmental measures from three cross sections of the Collaborative Perinatal Project (CPP), a panel spanning 7 years of children's lives beginning with pregnant women recruited at university hospitals between 1959 and 1965. Our outcome variables are the same six CPP measures of children's neurocognitive development that the earlier study examined [10], two measures at each follow-up age: 8 mo, 4 y, and 7 y. In order, these include the Bayley Mental and Motor Scales for Infant Development; the Stanford Binet Intelligence Scale Form L-M and the Graham-Ernhart Block Sort Test; and the Wechsler Intelligence Scale for Children (WISC) Full Scale IQ and the Wide Range Achievement Test (WRAT) of Reading. In our CPP data, drawn from the enhanced electronic datasets distributed by the Johns Hopkins School of Public Health, the WRAT scores are raw rather than normed, with a mean around 35 rather than 100. We found no qualitative differences between our results using the raw scores and those of the previous study [10], which used normed scores, when keeping the list of covariates fixed.

Data on physical health and neurocognitive characteristics were collected throughout the CPP panel, but detailed socioeconomic and other family characteristics were only collected at registration and again at 7 years. To our knowledge, no data were collected on time use among mothers or fathers in the CPP. Our covariates primarily consist of characteristics of the child and family measured at or before the child's birth. These include the sex, gestation weeks, and birth weight of the child; the child's total number of older siblings; the age, race/ethnicity, years of education, marital status, and mental health history of the mother at the time of birth; the age, years of education, and mental health history of the father at the time of birth; and the household's socioeconomic index at the time of registration. The last is the average of three percentile ranks: of the education of the household head, of the average income and education associated with the occupation of the head, and of family income. While socioeconomic characteristics were measured again at 7 years, we use characteristics measured at birth in our models for four reasons. First, results do not change appreciably when we include contemporaneous paternal education or family socioeconomic index; second, data on fathers is more sparse at the 7 year follow-up, which reduces sample size; third, the previous study that we revisit also uses characteristics at birth as covariates [10]; and fourth, theory suggests that child development should depend on past as well as current inputs.

We also use other covariates that were measured more or less contemporaneously. These include the exact age in months of the child at the time the neurocognitive test was administered, and the total number of younger siblings born by the time the child reached 7 years old. The ages of younger siblings were not recorded at 7 years, so we cannot extrapolate the number of living younger siblings at earlier waves. Instead, we specify younger siblings at age 7 as a covariate of child outcomes at each age: 8 mo, 4 y, and 7 y.

Compared to the study we are replicating [10], our complete list of covariates includes 5 new variables that we believe are likely to be important: the child's birth weight, the mother's and father's education levels at the time of birth, and counts of the child's older and younger siblings. The addition of these covariates reduces our sample size and thus our statistical power by only a small amount, as shown in Table 1. As discussed elsewhere [18], [19], the CPP included almost 60,000 live births, as shown in the top row. A large number of these babies, almost 13,000, had fewer than 37 weeks' gestation or no gestation data. Fewer than 700 were non-singleton births. Nearly 11,000 of the remaining records included no data on the father's age, cutting the sample to 34,914. Requiring all the basic covariates used in the earlier study [10] cuts the sample to 33,188, and requiring the additional 5 covariates we examine here further reduces it to 31,346. Not all of these children participated in data collection during the panel, lowering sample sizes in the outcome regressions to between 22,500 and 26,500.

**Table 1:** Children and covariates in the CPP sample.

	n
All children in the master dataset	59,392
Children with 37+ weeks gestation	59,392
All singleton births with 37+ weeks gestation	46,080
Singletons with 37+ and father's age	34,914
Singletons with 37+ and all basic covariates	33,188
Singletons with 37+, basic covariates and:	
Mother's education	33,091
Father's education	31,478
Birth weight	33,129
Older siblings	33,177
Younger siblings	33,188
All 5 of these	31,346

Source: Collaborative Perinatal Project (CPP).

Notes: “Basic covariates” include sex, gestation weeks, mother's race, parental ages at birth, marital status, parental history of mental illness, and the family socioeconomic index. Observations missing gestation and twin data are dropped successively in rows 2 and 3.

### Statistical Methods

The earlier study whose results we seek to revisit [10] modeled nonlinear partial relationships between maternal age or paternal age and child outcomes in equation (1) using a generalized additive model (GAM). All other covariates were constrained to have standard linear effects. We first replicated the earlier results [10] using the same software and methods, a GAM estimated using the *mgcv* library in *R*. We also conducted secondary analyses in *Stata* using ordinary least squares.

## Results

### Sample characteristics and correlations

Table 2 displays summary statistics for the six endogenous measures of children's neurocognitive outcomes and eight covariates of interest. There is no clear pattern between mean neurocognitive scores and their standard deviations; each measure has its own unique variance structure and coefficient of variation. Correlations between these six measures (not shown) reveal positive associations between measures taken at a particular age, and somewhat less correlation in measures across time for a particular child.

**Table 2:** Summary statistics of outcomes and key covariates.

	Mean	Standard Deviation	n
Bayley Mental	80.0	(5.4)	26,527
Bayley Motor	33.7	(4.5)	26,529
Stanford Binet Intelligence Scale	100.1	(16.8)	22,822
Graham Ernhart	34.6	(8.2)	22,523
WISC Full Scale IQ	98.5	(14.9)	23,717
WRAT Reading	37.4	(12.5)	23,604
Father's age, y	28.2	(6.8)	31,346
Mother's age, y	24.7	(5.8)	31,346
Father's education, y	11.2	(3.1)	31,346
Mother's education, y	11.0	(2.6)	31,346
Family socioeconomic index	52.7	(21.2)	31,346
Older siblings	1.9	(2.1)	31,346
Younger siblings	0.8	(1.1)	31,346
Birth weight, g	3,266.8	(489.4)	31,346

Source: Collaborative Perinatal Project (CPP).

As shown in the bottom half of the table, a difference of 3.5 y separates the average mother's age from the average father's age in the CPP. This gap has narrowed somewhat in the US over time; CDC natality statistics from 2006 show an average parental age gap of 2.7 y for first births [20]. This gap is not reflected in the average education levels in the CPP, which are only 0.2 year apart and not significantly different when observed independently.

There is substantial variation within the sample in the socioeconomic index, which by definition has an average around 50. Reflecting considerably higher total fertility rates around the time of the study, children in the CPP had an average of 2.7 siblings, 1.9 older plus 0.8 younger by the age of 7. This is consistent with the much higher Total Fertility Rate prevailing at the time of the CPP, 3.65 in 1960, versus about 2.0 to 2.1 today [21]. Children in the CPP were born at an average weight of 3,266.8 grams, which is roughly the same as the average birth weight today. In our sample, which conditions on 37 or more weeks' gestation in this sample, we found that 5% of babies were born under 2,500 grams (not shown), a common threshold definition of low birth weight.

Table 3 shows a matrix of Pearson correlation coefficients between the eight covariates shown in Table 1. The Pearson correlation is primarily sensitive to linear relationships, but it is a widely used and useful indicator of the covariance structure of the data. Mother's age and father's age are relatively more tightly correlated (Pearson correlation = 0.7980) than are mother's and father's education (0.6304). Differences within mother/father couples both in age and in years of education are statistically significant (not shown), but for separate reasons. Table 2 shows the average difference in age is large, and Table 3 shows it varies relatively little across couples. By comparison, the average difference in education levels is much smaller, but because there is less covariance between them, the variance in the difference is also smaller, so the difference is still statistically significant.

**Table 3:** Correlations between key covariates (*n* = 31,346).

	Father's age, y	Mother's age, y	Father's education, y	Mother's education, y	Family socioeconomic index	Older siblings	Younger siblings	Birth weight, g
Father's age, y	1.0000							
Mother's age, y	0.7980	1.0000						
Father's education, y	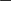 0.1607	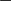 0.0772	1.0000					
Mother's education, y	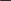 0.1123	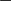 0.0272	0.6304	1.0000				
Family socioeconomic index	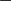 0.0302	0.0548	0.7918	0.5678	1.0000			
Older siblings	0.5255	0.5747	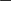 0.3078	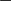 0.2870	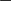 0.2458	1.0000		
Younger siblings	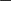 0.2001	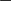 0.2400	0.0026	0.0093	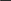 0.0344	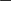 0.1456	1.0000	
Birth weight, g	0.0574	0.0845	0.0682	0.0704	0.1027	0.0695	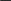 0.0013	1.0000

Source: Collaborative Perinatal Project (CPP).

Notes: All correlations are significant at the 5% level except between (1) younger siblings and father's education, (2) younger siblings and mother's education, and (3) birth weight and younger siblings.

Mother's and father's age are both negatively correlated with mother's and father's education (Pearson correlations of 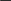
0.0272 to 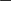
0.1607), while they both are positively correlated with the number of older siblings (0.5747 and 0.5255), and negatively with younger siblings (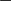
0.2400 and 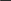
0.2001). Parental education is negatively correlated with older siblings (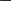
0.3078 and 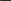
0.2870) but uncorrelated with younger siblings. The family socioeconomic index is indeed correlated with parental education (0.7918 and 0.5678), but the latter two variables appear to be measuring distinct characteristics. Birth weight, an indicator of the child's health endowment, is positively but only marginally correlated with the other six variables, most tightly with the socioeconomic index (0.1027). Because of this covariance structure, estimates of the marginal effects in equation (1) will be subject to omitted variable bias unless all the relevant 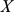
's and 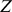
's are included.

### Model results

We next modeled the six outcomes variables using several GAMs with sequentially longer lists of covariates. We begin with the same Model 1 as specified in the earlier paper [10], in which the outcome variable is a nonlinear function of mother's and father's age, and a linear function of the child's age, gestation weeks, the child's sex, and the mother's race or ethnicity. Model 2 adds in indicator variables for the mother's marital status, the family's socioeconomic index, and two indicator variables for the mother's and father's past mental illness. Our unique contribution is Model 3, to which we have added mother's and father's education in years, numbers of older and younger siblings, and birth weight, all of which have linear effects.

**Figure 1:**
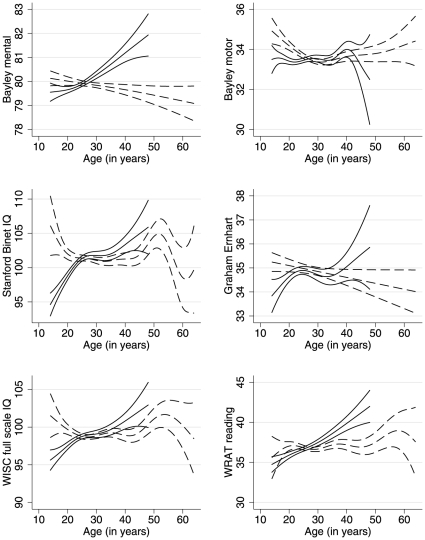
Partial predictions of the six outcome variables for maternal or paternal age. Predictions from Model 3, which adjusts for parental ages; age, sex, and gestation weeks of the child, mother's race or ethnicity and marital status, family socioeconomic index, parental history of mental illness, parental education, numbers of older and younger siblings, and birth weight. Solid lines for maternal age, dashed lines for paternal age, each showing mean and 95% confidence intervals. Nonlinear models fit using GAMs via *mgcv* in *R*.

We proceed to examine the nonlinear model results in the same way as the earlier study [10], but we caution that nonlinear results can be challenging to interpret. In Table 4 we report approximate *p*-values from tests of the statistical significance of the nonlinear effects of mother's and father's age, all generated by *mgcv*. Parental ages are most significant in Model 1, which includes the fewest covariates and also produces the lowest adjusted *R*-squared. As more covariates are added, parental age and specifically paternal age tends to lose significance while the adjusted *R*-squared, an index of the model's fit, improves monotonically. Separate results using the Akaike Information Criterion, another statistic that is commonly used to guide model selection, also reveal that model fit improves when the covariate list is expanded to include parental education. In Model 3, paternal age becomes insignificant for the Graham Ernhart and the WRAT Reading scores (*p*-values of 0.052 and 0.131). Paternal age remains statistically significant for the remaining four outcomes in these nonlinear models, but as Figure 1 reveals, this result is largely misleading.

**Table 4:** Summary of nonlinear models 1, 2, and 3.

		Model 1			Model 2			Model 3		
	Sample Size	Influence of Maternal Age *p*-value	Influence of Paternal Age *p*-value	Adjusted R-squared (%)	Influence of Maternal Age *p*-value	Influence of Paternal Age *p*-value	Adjusted R-squared (%)	Influence of Maternal Age *p*-value	Influence of Paternal Age *p*-value	Adjusted R-squared (%)
Bayley Mental 26,503	0.000	0.000	0.023	0.000	0.003	0.027	0.000	0.024	0.042	
Bayley Motor 26,505	0.897	0.000	0.060	0.243	0.000	0.070	0.061	0.000	0.090	
Stanford Binet Intelligence Scale 22,777	0.000	0.000	0.188	0.000	0.000	0.269	0.000	0.002	0.303	
Graham Ernhart 22,490	0.000	0.000	0.085	0.000	0.003	0.106	0.006	0.052	0.116	
WISC Full Scale IQ 22,811	0.000	0.000	0.185	0.000	0.000	0.285	0.000	0.003	0.329	
WRAT Reading 22,743	0.000	0.000	0.125	0.000	0.000	0.217	0.000	0.131	0.262	

Source: Collaborative Perinatal Project (CPP).

Notes: Model 1 includes the following covariates: mother's age, father's age, child's age, getstation weeks, child's sex, mother's race/ethnicity. Model 2 includes those covariates plus mother's marital status, family's socioeconomic index, and mother's and father's past mental illness (indicator variables). Model 3 includes those covariates plus mother's and father's education, total number of siblings, and birth weight.

Figure 1 plots partial predictions and their 95% confidence intervals for the six outcome variables based on Model 3 when varying just maternal age (solid lines) or just paternal age (dashed lines). Slopes in these graphs are the closest equivalent to a regression coefficient or marginal effect from standard linear analysis. Compared to previously published results [10], these partial predictions are less supportive of a negative relationship between paternal age and child outcomes. In four of the panels, the nonlinear association of the neurocognitive outcome with paternal age flattens out for a wide range around the sample mean of 28 y, revealing essentially no relationship. In the remaining two plots, of the Bayley Mental Scale at upper left and the Graham Ernhart Block Sort Test score at middle right, outcomes appear to decline linearly with father's age, but Table 4 reveals that only the former is statistically significant. The Bayley Motor Scale at upper right falls with father's age but only until age 30, after which it has an imprecise but non-negative effect. About 30% of the sample has fathers over 30.

In Table 5, we report selected regression coefficients and their standard errors from linear versions of Models 1, 2, and 3, estimated with ordinary least squares. Although Models 1 and 2 may be misspecified for certain outcomes when associations are restricted to be linear, Figure 1 suggests that the expanded covariate set in Model 3 tends to eliminate nonlinearities except at the extremes of parental ages. Imposing linearity across all models facilitates easier comparisons and helps reveal omitted variable bias.

**Table 5:** Comparison of select marginal effects in linear versions of models 1, 2, and 3.

Dependent Variable:				Dependent Variable:			
Bayley Mental	Model 1	Model 2	Model 3	Bayley Motor	Model 1	Model 2	Model 3
Mother's age, y	0.040***	0.031***	0.070***	Mother's age, y	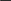 0.012	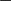 0.023***	0.005
	(0.009)	(0.009)	(0.010)		(0.008)	(0.008)	(0.009)
Father's age, y	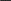 0.033***	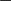 0.024***	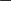 0.019**	Father's age, y	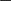 0.034***	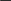 0.024***	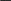 0.015**
	(0.008)	(0.008)	(0.008)		(0.007)	0.007	(0.007)
Socioeconomic index		0.016***	0.020***	Socioeconomic index		0.021***	0.015***
		(0.002)	(0.003)			(0.001)	(0.002)
Mother's education, y			0.010	Mother's education, y			0.035**
			(0.018)				(0.014)
Father's education, y			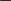 0.098***	Father's education, y			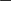 0.017
			(0.019)				(0.016)
Older siblings			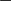 0.274***	Older siblings			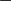 0.219***
			(0.023)				(0.019)
Younger siblings			0.050	Younger siblings			0.026
			(0.031)				(0.027)
Birth weight, g			0.001***	Birth weight, g			0.001***
			(0.000)				(0.000)

Source: Collaborative Perinatal Project (CPP). Notes: All three models specify linear relationships between the dependent and independent variables and are estimated by ordinary least squares. Robust standard errors are in parentheses, and asterisks denote statistical significance at the 1% (***), 5% (**), and 10% (*) level. Model 1 includes the following covariates: mother's age, father's age, child's age, getstation weeks, child's sex, mother's race/ethnicity. Model 2 includes those covariates plus mother's marital status, family's socioeconomic index, and mother's and father's past mental illness (indicator variables). Model 3 includes those covariates plus mother's and father's education, number of older siblings, number of younger siblings (at 7 years) and birth weight.

Table 5 shows that sequentially adding covariates across models attenuates the marginal effect of father's age, to the point of insignificance in the four outcomes past 8 mo. The coefficient on father's age in Model 3 remains negative and significant at the 5% level for the Bayley Mental and Motor Scales, but it has fallen in size by one third to one half (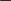
0.03 to 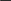
0.02 or 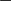
0.015). At 4 y, the coefficient on father's age turns positive and insignificant for the Stanford Binet, while for the Graham Ernhart model it falls by more than half and is significant only at the 10% level. At 7 y, neither the WISC IQ nor the WRAT Reading score vary significantly with father's age in Model 3, and both point estimates of the marginal effect are positive.

By contrast, adding covariates can raise or lower the marginal effect of mother's age, which remains positively associated with four of the six outcome measures in Model 3. Meanwhile, coefficients on the new covariates are almost always significant, especially for development at later ages. In Model 3, mother's education is positive and highly significant for all but the Bayley Mental Scale. The marginal effect of years of father's education is positive and significant for the four outcomes at 4 and 7 y, but it is also significantly smaller than the marginal effect of mother's education. The presence of older siblings has a negative and highly significant effect on 5 of the 6 outcomes, while younger siblings are also harmful for outcomes at 4 y and 7 y. Birth weight is always highly significant at the 1% level and positively associated with all six neurocognitive outcomes.

## Discussion

Broadly speaking, our results suggest an important role in the neurocognitive development of children played by family characteristics, and by extension assortative mating. We find that mother's education and the number of siblings are key variables in child outcomes, and to a lesser extent so too is father's education. That mother's education has a larger marginal effect than father's education suggests that the former reflects more than just heredity, because both parents contribute genetic endowments [14]. Because it is a good proxy of endowed health, birth weight is also a highly significant variable in explaining development, but its effect is largely independent from those of other covariates because birth weight is nearly orthogonal to most of them.

Without controlling for these variables, models of neurocognitive development will yield biased estimates of the marginal impact of correlated variables. The omission of mother's or father's education, both of which have a positive effect on child outcomes but are negatively correlated with father's age in the CPP data, produces an artificially large negative coefficient on father's age. The omission of number of siblings, which proxies for lower investments per child and thus has a negative effect on child outcomes, adds to the problem because it is positively associated with father's age.

The omitted variable bias plaguing earlier results on child neurocognitive outcomes [10] can be traced to a handful of demographic factors. In the CPP sample, older fathers seem to have paired with less educated mothers, as shown in Table 3, and their children's outcomes were lower as a result. In addition, older fathers in the CPP are themselves less educated, which may explain why they married less educated women. Educational disparities across parental birth cohorts could also be due to differences in the access to and price of education over time as states' educational policies evolved rapidly after the Second World War [22]. Finally, children in the CPP with older fathers and mothers typically also had more older siblings, leading to reductions in parental investments per child.

While our results significantly diminish the earlier findings regarding the marginal effect of father's age on children's neurocognitive outcomes in the CPP [10], they fall short of universally refuting them. There are traces of negative influences on two or three of our six outcomes, but we find these results not very compelling. Figure 1 shows that nonlinear estimates of outcomes using Model 3 often have U-shaped relationships with paternal age, which may be statistically significant per Table 4 but not indicative of a clear negative relationship. In the case of the Bayley Motor Scale, a GAM recovers a negative marginal effect of father's age only before 30 and basically no relationship for the 30% of the sample with fathers over age 30. From a biological perspective, it is unclear why advancing paternal age should be bad for the children of younger but not older fathers, or what it means. We suspect that yet another omitted variable may be generating this result for fathers under 30, because it does not fit the biological argument well. For other outcome variables, nonlinear results are either even more convoluted, hovering around a zero average effect, or they reveal an underlying relationship that is linear. In the linear version of Model 3 in Table 5, paternal age is negatively associated at the 5% level with the two neurocognitive measures at 8 mo, the Bayley Mental and Motor Scales (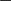
0.019 and 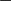
0.015). But at older ages, only the Graham-Ernhart Block Sort Test is negatively correlated with father's age (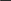
0.025), and only at the 10% level.

As shown in Table 4, the effects of father's age are greatly attenuated by the inclusion of expanded covariates, especially mother's education. In the case of the Graham Ernhart, the coefficient falls from 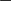
0.066 in Model 1 to 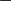
0.037 in Model 2 and finally to 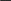
0.025 in Model 3, a reduction of more than half. To place this result in context, it is useful to examine the relative impact of the father's age coefficient. Table 5 shows that an additional year of father's age lowers the Graham-Ernhart score by 0.025, while an additional year of mother's education raises it by 0.303, or by more than an order of magnitude. Although these are technically cross-sectional estimates of marginal effects, we can translate them into longitudinal trends to provide a rough guess of the possible implications of delayed childbearing, as the original study [10] had also done. As stated earlier, the average maternal age at first birth has risen roughly 0.1 y each year, while the age gap between mothers and fathers has actually narrowed slightly. Meanwhile, the average years of education among mothers rose from 10.7 in the CPP around 1960, as shown in Table 1, to about 13.1 in CDC data from 2006 [20], an average annual increase of about 0.05 y. Assuming father's age has also increased 0.1 y each year, the total net effect on the Graham-Ernhart score from annual increases in father's age and mother's education would be an increase each year of about 0.01. Even the largest point estimates of the marginal effect of advancing paternal age in Table 3, those from Model 1, would not even net out to zero against the protective effects of increasing maternal education. This broader view suggests that the net effect of delayed childbearing on children's neurocognitive outcomes is likely to be beneficial on average, because it is paired with increasing female education, which is robustly protective and appears to stem at least in part from nurturing influences.

It is worth reiterating that in the linear Model 3, shown in Table 5, evidence for a negative effect of father's age on development is strongest in the case of the two indicators measured at 8 mo. If mother's education is highly beneficial because it proxies for higher quality and quantity of time inputs, one might expect its impact to rise with treatment intensity, for which the age of the child is a proxy. If advanced paternal age has deleterious effects on child outcomes through a biological channel, one might find such effects starting from birth. It is plausible that nurturing elements associated with mother's education may increasingly offset the negative effects associated with paternal age over the life of the child. We see some suggestive evidence of this in Table 5.

Our findings also speak to the use and misuse of nonlinear modeling when there may be omitted variables. The latter can generate spurious nonlinear effects of included variables that may not represent anything causal or very meaningful. We expect deleterious biological influences such as sperm mutation to increase along with paternal age and thus reduce child outcomes, but there are many other environmental factors that are also related to paternal age and also affect child outcomes. In particular, parental education is positively correlated with child outcomes because it typically proxies the quality of parental investments in the child. It is positively correlated with age within a given cohort of parents, but within a cross section of parents at many ages, older parents are likely to have lower education because average educational attainment has been increasing over time. Thus omitting parental education will induce a spurious nonlinear relationship between child outcomes and father's age, one that rises at first, reflecting greater educational attainment among parents aged 25 y compared with those aged 16 y, and then falls because parents aged 45 y have less education by simply having been born earlier.

Hypothesis testing can be difficult in a nonlinear environment, as revealed by the relatively misleading statistical significance tests in Table 4. Nonlinear modeling may be more appealing in a forecasting context, in which we are often more concerned about overall model fit and prediction than about inference and hypothesis testing. A low *p*-value on a variable in a nonlinear framework is a more sufficient condition when the the size and sign of its marginal effect are not so important to the bottom line. But our results indicate that when we are concerned with specific pathways between child outcomes and natural and nurturing influences, it is critical to examine size and sign and to control for a broad array of parental and family characteristics when drawing inferences. In the case of children's neurocognitive outcomes, we find that mother's education and family size seem to matter much more than paternal age per se.
